# Interventions to Reduce Risk for Pathogen Spillover and Early Disease Spread to Prevent Outbreaks, Epidemics, and Pandemics

**DOI:** 10.3201/eid2903.221079

**Published:** 2023-03

**Authors:** Neil M. Vora, Lee Hannah, Chris Walzer, Mariana M. Vale, Susan Lieberman, Ashley Emerson, Jonathan Jennings, Robyn Alders, Matthew H. Bonds, Jo Evans, Bhavana Chilukuri, Sonila Cook, Nigel C. Sizer, Jonathan H. Epstein

**Affiliations:** Conservation International, Arlington, Virginia, USA (N.M. Vora, L. Hannah);; University of Veterinary Medicine Savoyenstr, Vienna, Austria (C. Walzer);; Wildlife Conservation Society, Bronx, New York, USA (C. Walzer, S. Lieberman);; Universidade Federal do Rio de Janeiro, Rio de Janeiro, Brazil (M.M. Vale);; Health in Harmony, Portland, Oregon, USA (A. Emerson, J. Jennings);; Australian National University, Canberra, Australian Capital Territory, Australia (R. Alders);; Chatham House Global Health Programme, London, UK (R. Alders);; Harvard Medical School, Boston, Massachusetts, USA (M.H. Bonds);; PIVOT, Ifanadiana, Madagascar (M.H. Bonds);; Dalberg Catalyst, Washington DC, USA (J. Evans, B. Chilukuri, S. Cook, N.C. Sizer);; EcoHealth Alliance, New York, NY, USA (J.H. Epstein)

**Keywords:** zoonoses, vector-borne infections, bioterrorism and preparedness, public health, pandemic prevention, pandemic preparedness, One Health, policy, planetary health

## Abstract

The pathogens that cause most emerging infectious diseases in humans originate in animals, particularly wildlife, and then spill over into humans. The accelerating frequency with which humans and domestic animals encounter wildlife because of activities such as land-use change, animal husbandry, and markets and trade in live wildlife has created growing opportunities for pathogen spillover. The risk of pathogen spillover and early disease spread among domestic animals and humans, however, can be reduced by stopping the clearing and degradation of tropical and subtropical forests, improving health and economic security of communities living in emerging infectious disease hotspots, enhancing biosecurity in animal husbandry, shutting down or strictly regulating wildlife markets and trade, and expanding pathogen surveillance. We summarize expert opinions on how to implement these goals to prevent outbreaks, epidemics, and pandemics.

The pathogens that cause most emerging infectious diseases in humans originate in animals, particularly wildlife, and then spill over into humans. Emerging infectious diseases, including pandemic influenza, Ebola, mpox, and HIV/AIDS, have had profound effects on humanity (*1*). SARS-CoV-2, the virus that causes COVID-19, also likely emerged in humans through spillover (*2*,*3*). Land-use change, animal husbandry, and commercial wildlife markets and trade create opportunities for spillover, and climate change is further increasing the risk for infectious disease emergence (*4*).

The ecologic disruption caused when land with intact ecosystems is converted for purposes such as agriculture increases contact between humans, domestic animals, and wildlife, providing opportunities for spillover. Among ecosystem types, clearing and degradation of tropical and subtropical forests likely carries the highest risk for spillover (*5*,*6*). Forest clearing and degradation brings humans to the forest edge, increasing opportunities for humans and domestic animal contact with wildlife and subsequent pathogen transmission (*6*–*9*). Forest clearing and degradation also causes loss of biodiversity, which disrupts and decreases natural species assemblages and favors animals that can survive near humans, which often are animals associated with zoonotic pathogens, such as bats and rodents (*10*). In addition, those wildlife species might experience physiologic stress from habitat disruption, increasing their risk of becoming infected with or shedding viruses (*8*). Furthermore, commercial exploitation of wildlife through markets and trade often increases when previously intact forests are opened (*11*). The subsequent admixture of wildlife and domestic animals creates further opportunities for spillover and potential for viral recombination.

Spillover events combined with the interconnectedness of the modern world will accelerate the frequency of outbreaks, epidemics, and pandemics unless underlying spillover drivers are addressed (*12*). Unfortunately, spillover prevention has largely been sidelined in discussions on reforming global approaches to pandemics during the rebuilding from COVID-19 (*13*). Instead, most discourse, often couched as prevention, focuses on postspillover interventions, such as outbreak response, health system strengthening, and vaccine development (*14*,*15*). Such interventions are essential but are not prevention; on their own, postspillover interventions are insufficient to safeguard against the catastrophic threats posed by pandemics. Even when outbreaks are rapidly identified, postspillover interventions can be unsuccessful. Furthermore, timely and effective vaccines and therapeutics might not be available for every future disease. In addition, vaccine and therapeutic effectiveness at the population level can be hampered by supply and distribution challenges, especially in low-income countries, and limited uptake resulting from disinformation and personal beliefs (*16*).

All of those factors point to the urgent need to invest in interventions to prevent spillover and to curb early disease spread among domestic animals and humans. Doing so would be consistent with One Health, an integrated, unifying approach to sustainably balance and optimize the health of humans, animals, and ecosystems (*17*).

We summarize expert opinions on how interventions to reduce risk for spillover and curb early disease spread can be implemented. The interventions we describe fall into 5 categories: stop clearing and degradation of tropical and subtropical forests, improve health and economic security of communities living in emerging infectious disease hotspots, enhance biosecurity in animal husbandry, shut down or strictly regulate wildlife markets and trade, and expand pathogen surveillance at interfaces between humans, domestic animals, and wildlife.

## Stop Clearing and Degradation of Tropical and Subtropical Forests

Multiple approaches can be used to address spillover caused by tropical and subtropical forest clearing and degradation. Integrated policies with increased enforcement aim to remove incentives for deforestation while respecting rights of Indigenous Peoples and local communities (IPLCs). Brazil successfully demonstrated this approach in the Amazon Basin. In 2004, the government of Brazil, working with international donors, provided sufficient funds to put into action policies that resulted in a 70% reduction in annual deforestation (*18*). After 2012, policy enforcement weakened and deforestation rose, demonstrating that sustained commitment is crucial for success. Agricultural production increased while the policy was in effect, suggesting reduced deforestation did not affect economic development (*18*–*20*). Of note, nearly 50% of intact forests in the Amazon Basin are in Indigenous territories, and deforestation rates on these lands are lower than elsewhere (*21*).

Regulatory and market-based measures can establish standards that affect international supply and demand for products and lead to decreased deforestation. For instance, Europe is considering restricting imports of commodities such as soybeans, beef, cocoa, and palm oil if production is linked to deforestation (*22*). In an example of market-based measures, >30 financial institutions with trillions of US dollars in assets have pledged to end investment in activities linked to deforestation (*23*).

Payment for ecosystem services gives landholders incentives to maintain or increase forest cover on their lands, thus enhancing levels of ecosystem services provided by those forests (*24*). Success of this strategy has varied across implementation sites; better outcomes might be achieved by focusing on local or regional scales, using in-kind contributions rather than cash payments, and emphasizing equity (*24*).

Community-designed interventions provide local services, such as healthcare, to IPLCs living within and near forests to minimize reliance on deforestation to generate income. For example, communities in Indonesian Borneo were heavily engaged in local illegal logging partly because of high healthcare costs (*25*). When supported to design their own solutions, IPLCs chose to build a medical center and start an alternative livelihood program (*25*). After those solutions were put in place over a decade, at a total cost of US $5.2 million, the percentage of households relying on illegal logging decreased by 90%, forest regrowth increased by 21,000 hectares, and US $65 million in carbon loss was averted (*25*). Similar models have been successfully replicated in Madagascar and Brazil.

## Improve Health and Economic Security of Communities Living in Emerging Infectious Disease Hotspots

In many emerging infectious disease hotspots, little intact forest remains, and spillover events occur because dense human and domestic animal populations live closer to wildlife and commercial activities might involve intentional (e.g., wildlife trade) or unintentional contact with wildlife (*26*). Such areas make up only 4% of global area (10% of tropical area), but account for 60% of global spillover risk (*26*). Thus, community-designed interventions to decrease human and domestic animal contact with wildlife probably represent the best means to reduce virus spillover in these areas. Many communities in emerging infectious disease hotspots lack access to healthcare, sustainable livelihoods, food security, and education (*27*). Improving health and economic security are often high priorities for these communities, creating alignment between local needs and global pandemic prevention priorities.

Actions to reduce local risk for zoonotic diseases can be simple. For example, Nipah virus infection risk can be reduced by covering the shaved areas of palm tree trunks and sap vessels, thus preventing contamination with excrement of bats that feed on these palms (*28*). Where contact cannot be eliminated, improved practices, such as better sanitary practices during wildlife butchering, can minimize spillover (*29*).

Community-designed interventions to reduce human–wildlife contact and decrease spillover risk have not been widely implemented. However, projects in several countries, such as in Indonesia (as described in the previous section) and in Uganda, show that changing relationships between communities, forests, and wildlife are possible and beneficial (*25*,*30*). A project in Manombo Rainforest, Madagascar, aims to put in place community-designed conservation initiatives and collect metrics over 10 years to assess effects on biodiversity and wildlife and human health (A. Emerson et al., unpub. data). More projects specifically targeting spillover risk in emerging disease hotspots are needed as proofs of concept (*31*). Such pioneering projects should include measurement of spillover within target communities to enable thorough outcome assessments (*32*).

## Enhance Biosecurity in Animal Husbandry

The World Organisation for Animal Health (WOAH) defines biosecurity in animal husbandry as “a set of management and physical measures designed to reduce the risk of introduction, establishment and spread of animal diseases, infections or infestations to, from and within an animal population” (*33*). Several zoonotic viruses, including highly pathogenic avian influenza A(H5N1) virus, 2009 pandemic influenza A(H1N1) virus, Nipah virus, and Middle East respiratory syndrome coronavirus (MERS-CoV), have emerged from wildlife reservoirs into humans via commercial animal industries (*5**,**34*–*36*).

Improving biosecurity in animal husbandry requires management measures, such as quarantining new animals and vaccinating animals against endemic disease, and physical measures, such as enclosures that separate farm animals from each other and from wildlife (*37*). However, backyard flocks and large-scale commercial industrial systems face challenges in implementing biosecurity (*38*). Standard biosecurity recommendations are rarely tailored for extensive production systems in resource-limited settings, and translating recommendations into local languages requires time and knowledge of local perceptions of disease (*37*,*38*). Commercial industrial systems require major investments in biosecurity measures to counteract zoonotic disease transmission risks posed by raising a high density of genetically homogeneous, single-age animals, especially when animal welfare is suboptimal (*39*).

In addition to biosecurity challenges, humans also have major effects on disease incursion into and spread within backyard and commercial industrial systems. Underfunded animal health and extension services, which contributes to ineffective livestock disease surveillance, and chronic household food insecurity (i.e., lack of regular access to sufficient safe and nutritious food), drive risky husbandry practices and household food choices such as consumption of sick animals or animals that died of disease (*40*,*41*). Two global institutions mandated to oversee animal disease prevention and control recognize these challenges: the Food and Agriculture Organization of the United Nations (FAO) and WOAH. In April 2022, FAO and WOAH signed an agreement with the World Health Organization and the United Nations Environment Program to sustainably balance and optimize the health of humans, animals, plants, and the environment and released the One Health Joint Plan of Action to improve prevention, prediction, detection, and response to health threats (*42*,*43*). This partnership will require engagement and support from national and local agencies and organizations.

Biosecurity could be greatly enhanced through investments in public and private animal health services, identification of animal illnesses of public health concern by animal holders, and interventions to address these (*26*,*44*). Benefits of this approach include decreased pathogen transmission between wildlife and domestic animals, enhanced disease surveillance sensitivity via increased producer trust in services, fewer greenhouse gas emissions through efficiency, and improved food security (*37*,*40*,*45*,*46*). This approach is fully compatible with the described community-designed interventions and could be implemented by the same project teams, saving costs. Emerging infectious disease hotspots are among the highest priority areas for reducing spillover risk associated with animal husbandry. Therefore, community-designed interventions and animal husbandry projects should be integrated into any One Health pandemic prevention framework.

Controlling vaccine-preventable diseases in domestic animals also should be prioritized. Vaccination benefits communities and domestic animals through reduced illness and death from vaccine-targeted disease. Vaccination also reduces the spread of emerging infectious diseases because diagnosis can be delayed when emerging infectious diseases have similar clinical signs to vaccine-preventable diseases. For example, identification of highly pathogenic avian influenza in chickens frequently is delayed in areas with low Newcastle disease vaccination rates (*45*,*47*). Similarly, control for outbreaks of African swine fever (ASF), a disease with no effective vaccine, is hampered by low vaccination rates against classical swine fever (CSF) because the diseases have similar clinical signs, making ASF differentiation difficult in herds not vaccinated against CSF (*48*). In China, a 2019 pork shortage caused by ASF might have increased consumer demand for wildlife, thereby increasing the risk of pathogen spillover (*2*).

## Shut Down or Strictly Regulate Wildlife Markets and Trade

Legal and illegal wildlife markets and trade pose a high risk for emergence, amplification, and transmission of zoonotic pathogens, as observed with mpox virus, SARS-CoV-1, and probably SARS-CoV-2 (*2*,*3*,*5*). Wildlife trade is driven by demand for animals as food and pets and for skins, traditional medicines, and ornaments. For pandemic prevention, the primary focus should be live and freshly butchered wild birds and mammals because those pose the highest risk for spillover. Furthermore, prevention should focus on commercial wildlife markets and trade but should prioritize the rights of IPLCs, who are often dependent on wildlife for food and livelihoods. Addressing wildlife markets and trade that lead to pathogen spillover will require investments in 4 areas.

First, policy reform to restrict or close legal commercial wildlife markets and trade is needed. New legislation and amendments to existing laws should aim to end the sale of live and freshly butchered wild birds and mammals for commercial purposes; such reforms are consistent with recommendations released in 2021 by the World Health Organization (*49*). Several countries have taken these steps. For example, China banned farming, hunting, trading, and consuming terrestrial wildlife in the context of COVID-19 in February 2020 (*50*). In March 2020, Gabon banned sale and consumption of pangolins and bats (*51*).

Second, legislation and enforcement need to reflect efforts to address illegal wildlife markets and trade. Law enforcement, judicial, and other agencies mandated to stop illegal activities and bring criminals to justice should have budgets for staffing, equipment, training, enforcement, and judicial activities. In addition, they should have sufficient legal mandates to use all available investigative approaches and be held to the highest integrity standards to eradicate corruption. Although pathogen spillover is indifferent to the legality of individual animals in trade, increased vigilance to combat wildlife trafficking remains a priority.

Third, programs focusing on local communities and the rural poor need to be implemented to reduce dependence on wildlife markets and trade for income. Partnerships between local communities and governments will be crucial to provide incentives for robust and sustainable income streams that do not depend on wildlife markets and trade.

Fourth, to end the purchase and desirability of live wild birds and mammals, government leaders and experts need to lead behavior change efforts that apply behavioral science, psychology, economics, and social marketing best practices. Civil society and academia have capacity to find solutions. Several projects are underway, but more effective and sustainable efforts could benefit from public institutions leading or co-leading scale-up programs. In numerous countries, wildlife consumption, especially in urban centers, is not required for food security; rather, wildlife is consumed as a luxury, as a status symbol, or for perceived health reasons. In China, the price for wildlife is generally 2- to 5-fold higher than for pork, the most common animal protein source in the country, and even higher for exotic, endangered, or illegally obtained species (*52*,*53*). Reducing urban demand for wildlife will have a positive effect on IPLCs by reducing economic incentives to those supplying the trade and leaving more wildlife for IPLC subsistence needs. A strategy to ensure recognition and support of IPLC rights is vital, but that support must not be used as a smokescreen to continue business-as-usual in commercial markets and trade.

## Expand Pathogen Surveillance at Interfaces between Humans, Domestic Animals, and Wildlife

Pathogen surveillance platforms that include coordinated multisectoral efforts can increase their effectiveness (*54*). In this context, pathogen surveillance refers to various ongoing active and passive systems for collecting and reporting data on exposures or infections at the human or animal population level; the frequency and severity of disease caused by a given pathogen; and the evolution of microbes circulating in natural reservoirs and additional hosts (*55*). Integrated surveillance systems provide insights into zoonotic pathogens, such as Ebola virus, in their ever-changing natural reservoirs, as well as data on spillover into domestic animals and humans, which can inform targeted pandemic prevention, preparedness, and response strategies (*56*,*57*). For example, 25 years of data showed that Hendra virus spillover from bats to horses increased during periods of environmental stress; this finding provides evidence for using forest restoration as an ecologic countermeasure to reduce future spillovers (*58*).

Systematic wildlife surveillance will require substantial veterinary medical capacity and massive strengthening of multisectoral, decentralized, laboratory networks to support molecular and serologic screening of animal and human samples. Laboratory-based surveillance should include pathogen-specific molecular and serologic diagnostic assays and unbiased high-throughput screening tools (*59*,*60*). As whole-genome sequencing platforms and near real-time genomic sequencing for examining viral evolution and epidemiology become more common and field applicable, investments in bioinformatics capacity to analyze genomic data will become increasingly vital.

For humans, data can be extracted from several primary sources, including health information systems, sentinel surveillance sites, and repeated standard household surveys, to monitor for pathogen emergence. Such systems could be extended to wild and domestic animal populations. Many human febrile illnesses in low-income countries never reach the health system and are not thoroughly evaluated; thus, many viral infections are never diagnosed (*61*,*62*). Routine diagnostic systems for traditionally undiagnosed illnesses could be improved by increasing libraries of pathogens found in wild and domestic animal populations. However, limited access to diagnostic testing is not just confined to low-income countries; even countries like the United States experienced challenges that hampered disease control efforts during the COVID-19 pandemic and mpox epidemic (*63*,*64*).

Mounting evidence suggests that zoonotic pathogen spillover occurs more frequently than previously known. A study of exposure to batborne SARS-related coronaviruses suggested that ≈66,000 persons are infected with SARS-related coronaviruses annually in South and Southeast Asia (*65*). High rates of pre–COVID-19 SARS-related coronavirus exposure also were observed in Sierra Leone (*66*). This evidence underscores the need to improve surveillance for zoonotic pathogens that have pandemic potential.

## Discussion

COVID-19 and mpox exposed inadequacies in current domestic and global approaches to pandemics and their prevention. A narrow window of time for reform exists before the next crisis of zoonotic origin takes hold (*14*,*15*). History suggests that the most likely source of the next pandemic will be spillover of a novel virus from wildlife directly into humans or into domestic animals and then humans (*1*). Therefore, we must address underlying drivers of pathogen spillover to prevent outbreaks.

We have provided a nonexhaustive set of interventions to reduce risk of spillover. Pandemic prevention is a global imperative and must be addressed as such. Programs funded by high-income countries and focused on bilateral aid remain critical, but no country should carry the costs alone when the stakes are so high. Programs like the US Agency for International Development (USAID) Emerging Pandemic Threats PREDICT program (2009–2020) have transformed zoonotic disease prevention efforts by strengthening local workforce and laboratory capacity to detect and respond to known and unknown zoonotic viral threats (*67*). USAID continues to invest in spillover prevention in priority countries through projects to develop a One Health workforce via university networks in Asia and Africa and develop interventions to stop spillover at key high-risk human-animal interfaces. Despite the focus on spillover prevention, these programs are subject to changes in governmental priorities and are not funded at a scale that can sufficiently address the global threat. An integrated global pandemic prevention (prespillover) and preparedness (postspillover) strategy would cost US ≈$20–$50 billion annually from all sources (*14*,*68*). Published research indicates that the pandemic prevention (prespillover) initiatives we describe can be achieved for US ≈$20 billion per year globally, representing a fraction of the trillions of dollars, and millions of lives, lost from COVID-19 (*30*). 

Addressing the drivers of spillover will have benefits beyond pandemic prevention, including mitigating climate change, preventing biodiversity loss, enhancing basic human health, respecting human rights, and promoting sustainable development (*10*). Thus, as the World Bank Pandemic Fund and the World Health Assembly pandemic accord both take shape, the activities we describe must be included (*13*). The UN climate summit commitment of billions of dollars to help end deforestation in >100 countries by 2030 is promising (*23*), and health outcomes should be prioritized to maximize impacts and minimize the risk for failure seen with prior commitments. Irrespective of high-level governance agreements, compliance on the ground will require active engagement by communities that believe the proposed measures will make a positive difference in their lives.

In practice, One Health is often narrowly applied to public health approaches after spillover has occurred. To stop future waves of accelerating and intensifying outbreaks, epidemics, and pandemics, the global community needs to broaden One Health approaches and embrace initiatives to prevent spillover (*69*). Addressing the multitiered complexities of spillover events, nature, and human behavior will require stacked safeguards before and after the point of spillover to decrease the risk for future pandemics. Thus, the One Health approach for pandemics requires coordination, collaboration, and major new resources distributed across human health, animal health, environmental, and food safety agencies (*70*).

Preventing spillover is an issue of equity. Focusing only on postspillover interventions signals that the global community is tolerant of outbreaks in the most resource-limited settings, as long as those outbreaks do not grow into epidemics or pandemics. Preventing outbreaks will save lives in some of the world’s most vulnerable regions, which will help ensure equitably distributed health benefits.

A robust research agenda will be essential for examining causal links between the interventions we propose and subsequent spillover reductions. Available evidence suggests that these interventions will have meaningful effects on reducing the probability of spillover events (*5*). However, to date, few large-scale programs focus on spillover prevention and evaluate efficacy of interventions; more large-scale implementation is needed to build an evidence base to show how investments in addressing the drivers of spillover lead to lower spillover risk. Nonetheless, we already know enough to act now, and the Precautionary Principle (https://unesdoc.unesco.org/ark:/48223/pf0000139578) dictates that we must.

Zoonotic pandemic risk is heightened by humanity’s broken relationship with nature. The actions we describe will reduce the risk of spillover and early disease spread and address pandemics, climate change, biodiversity loss, and inequity. Evidence for these actions existed before COVID-19, but no action was taken. However, as we emerge from the acute phase of the current pandemic, we can take actions to prevent the next pandemic. We cannot undo the past, but we must do better in the future.

## References

[1] Jones KE, Patel NG, Levy MA, Storeygard A, Balk D, Gittleman JL, et al. Global trends in emerging infectious diseases. Nature. 2008;451:990–3. 10.1038/nature06536 18288193 PMC5960580

[2] Lytras S, Xia W, Hughes J, Jiang X, Robertson DL. The animal origin of SARS-CoV-2. Science. 2021;373:968–70. 10.1126/science.abh0117 34404734

[3] Worobey M, Levy JI, Malpica Serrano L, Crits-Christoph A, Pekar JE, Goldstein SA, et al. The Huanan Seafood Wholesale Market in Wuhan was the early epicenter of the COVID-19 pandemic. Science. 2022;377:951–9. 10.1126/science.abp8715 35881010 PMC9348750

[4] Carlson CJ, Albery GF, Merow C, Trisos CH, Zipfel CM, Eskew EA, et al. Climate change increases cross-species viral transmission risk. Nature. 2022;607:555–62. 10.1038/s41586-022-04788-w 35483403

[5] Alimi Y, Bernstein A, Epstein J, Espinal M, Kakkar M, Kochevar D, et al. Report of the scientific task force on preventing pandemics [cited 2021 Nov 1]. https://cdn1.sph.harvard.edu/wp-content/uploads/sites/2343/2021/08/PreventingPandemicsAug2021.pdf

[6] Daszak P, Amuasi J. das Neves CG, Hayman D, Kuiken T, Roche B, et al.; Intergovernmental Science-Policy Platform on Biodiversity and Ecosystem Services. Workshop report on biodiversity and pandemics.

[7] Loh EH, Zambrana-Torrelio C, Olival KJ, Bogich TL, Johnson CK, Mazet JA, et al. Targeting transmission pathways for emerging zoonotic disease surveillance and control. Vector Borne Zoonotic Dis. 2015;15:432–7. 10.1089/vbz.2013.1563 26186515 PMC4507309

[8] Plowright RK, Reaser JK, Locke H, Woodley SJ, Patz JA, Becker DJ, et al. Land use-induced spillover: a call to action to safeguard environmental, animal, and human health. Lancet Planet Health. 2021;5:e237–45. 10.1016/S2542-5196(21)00031-0 33684341 PMC7935684

[9] Faust CL, McCallum HI, Bloomfield LSP, Gottdenker NL, Gillespie TR, Torney CJ, et al. Pathogen spillover during land conversion. Ecol Lett. 2018;21:471–83. 10.1111/ele.12904 29466832

[10] Keesing F, Ostfeld RS. Impacts of biodiversity and biodiversity loss on zoonotic diseases. Proc Natl Acad Sci U S A. 2021;118:e2023540118. 10.1073/pnas.2023540118 33820825 PMC8092607

[11] United Nations Office on Drugs and Crime. Combating wildlife and forest crime. Global Pramme for Combating Wildlife and Forest Crime annual report 2020 [cited 2021 Nov 1]. https://www.unodc.org/documents/Wildlife/Annual_Report_GPWLFC2020.pdf

[12] Marani M, Katul GG, Pan WK, Parolari AJ. Intensity and frequency of extreme novel epidemics. Proc Natl Acad Sci U S A. 2021;118:e2105482118. 10.1073/pnas.2105482118 34426498 PMC8536331

[13] Vora NM, Hannah L, Lieberman S, Vale MM, Plowright RK, Bernstein AS. Want to prevent pandemics? Stop spillovers. Nature. 2022;605:419–22. 10.1038/d41586-022-01312-y 35551284

[14] G20 High Level Independent Panel. A global deal for our pandemic age [cited 2021 Nov 1]. https://pandemic-financing.org/report/foreword

[15] Sirleaf EJ, Clark H. Report of the Independent Panel for Pandemic Preparedness and Response: making COVID-19 the last pandemic. Lancet. 2021;398:101–3. 10.1016/S0140-6736(21)01095-3 33991477 PMC9751704

[16] Hotez P, Batista C, Ergonul O, Figueroa JP, Gilbert S, Gursel M, et al. Correcting COVID-19 vaccine misinformation: Lancet Commission on COVID-19 Vaccines and Therapeutics Task Force Members. EClinicalMedicine. 2021;33:100780. 10.1016/j.eclinm.2021.100780 33718854 PMC7935671

[17] World Health Organization. Joint Tripartite and UNEP Statement: Tripartite and UNEP support OHHLEP's definition of “One Health” 2021 Dec 1 [cited 2021 Dec 1]. https://www.who.int/news/item/01-12-2021-tripartite-and-unep-support-ohhlep-s-definition-of-one-health

[18] Nepstad D, McGrath D, Stickler C, Alencar A, Azevedo A, Swette B, et al. Slowing Amazon deforestation through public policy and interventions in beef and soy supply chains. Science. 2014;344:1118–23. 10.1126/science.1248525 24904156

[19] Nepstad D, Soares-Filho BS, Merry F, Lima A, Moutinho P, Carter J, et al. Environment. The end of deforestation in the Brazilian Amazon. Science. 2009;326:1350–1. 10.1126/science.1182108 19965742

[20] West TAP, Fearnside PM. Brazil’s conservation reform and the reduction of deforestation in Amazonia. Land Use Policy. 2021;100:105072. 10.1016/j.landusepol.2020.105072

[21] Food and Agriculture Organization of the United Nations. Forest governance by indigenous and tribal peoples [cited 2023 Jan 28]. https://www.fao.org/americas/publicaciones-audio-video/forest-gov-by-indigenous/en

[22] European Union. European Green Deal: Commission adopts new proposals to stop deforestation, innovate sustainable waste management and make soils healthy for people, nature and climate [cited 2023 Jan 28]. https://ec.europa.eu/commission/presscorner/detail/en/ip_21_5916

[23] Catanoso J. COP26 Glasgow Declaration: salvation or threat to Earth’s forests? Mongabay 2021 Nov 3 [cited 2021 Dec 1]. https://news.mongabay.com/2021/11/cop26-glasgow-declaration-salvation-or-threat-to-earths-forests

[24] Grima N, Singh SJ, Smetschka B, Ringhofer L. Payment for Ecosystem Services (PES) in Latin America: analysing the performance of 40 case studies. Ecosyst Serv. 2016;17:24–32. 10.1016/j.ecoser.2015.11.010

[25] Jones IJ, MacDonald AJ, Hopkins SR, Lund AJ, Liu ZY-C, Fawzi NI, et al. Improving rural health care reduces illegal logging and conserves carbon in a tropical forest. Proc Natl Acad Sci U S A. 2020;117:28515–24. 10.1073/pnas.2009240117 33106399 PMC7668090

[26] Allen T, Murray KA, Zambrana-Torrelio C, Morse SS, Rondinini C, Di Marco M, et al. Global hotspots and correlates of emerging zoonotic diseases. Nat Commun. 2017;8:1124. 10.1038/s41467-017-00923-8 29066781 PMC5654761

[27] Garchitorena A, Sokolow SH, Roche B, Ngonghala CN, Jocque M, Lund A, et al. Disease ecology, health and the environment: a framework to account for ecological and socio-economic drivers in the control of neglected tropical diseases. Philos Trans R Soc Lond B Biol Sci. 2017;372:20160128. 10.1098/rstb.2016.0128 28438917 PMC5413876

[28] Khan SU, Gurley ES, Hossain MJ, Nahar N, Sharker MAY, Luby SP. A randomized controlled trial of interventions to impede date palm sap contamination by bats to prevent nipah virus transmission in Bangladesh. PLoS One. 2012;7:e42689. 10.1371/journal.pone.0042689 22905160 PMC3414453

[29] Wolfe ND, Switzer WM, Carr JK, Bhullar VB, Shanmugam V, Tamoufe U, et al. Naturally acquired simian retrovirus infections in central African hunters. Lancet. 2004;363:932–7. 10.1016/S0140-6736(04)15787-5 15043960

[30] Bernstein AS, Ando AW, Loch-Temzelides T, Vale MM, Li BV, Li H, et al. The costs and benefits of primary prevention of zoonotic pandemics. Sci Adv. 2022;8:eabl4183. 10.1126/sciadv.abl4183 35119921 PMC8816336

[31] Bloomfield LSP, McIntosh TL, Lambin EF. Habitat fragmentation, livelihood behaviors, and contact between people and nonhuman primates in Africa. Landsc Ecol. 2020;35:985–1000. 10.1007/s10980-020-00995-w

[32] Salerno J, Ross N, Ghai R, Mahero M, Travis DA, Gillespie TR, et al. Human-wildlife interactions predict febrile illness in park landscapes of western Uganda. EcoHealth. 2017;14:675–90. 10.1007/s10393-017-1286-1 29181611

[33] World Organisation for Animal Health, Office of Innovation and Entrepreneurship. Terrestrial animal health code glossary 2018 [cited 2021 Nov 1]. https://www.oie.int/fileadmin/Home/eng/Health_standards/tahc/2018/en_glossaire.htm

[34] Brockwell-Staats C, Webster RG, Webby RJ. Diversity of influenza viruses in swine and the emergence of a novel human pandemic influenza A (H1N1). Influenza Other Respir Viruses. 2009;3:207–13. 10.1111/j.1750-2659.2009.00096.x 19768134 PMC2746644

[35] Gambotto A, Barratt-Boyes SM, de Jong MD, Neumann G, Kawaoka Y. Human infection with highly pathogenic H5N1 influenza virus. Lancet. 2008;371:1464–75. 10.1016/S0140-6736(08)60627-3 18440429

[36] Han H-J, Yu H, Yu X-J. Evidence for zoonotic origins of Middle East respiratory syndrome coronavirus. J Gen Virol. 2016;97:274–80. 10.1099/jgv.0.000342 26572912 PMC7087374

[37] Otte J, Rushton J, Rukambile E, Alders RG. Biosecurity in village and other free-range poultry—trying to square the circle? Front Vet Sci. 2021;8:678419. 10.3389/fvets.2021.678419 34150895 PMC8207203

[38] Bagnol B, Naysmith S, Bruyn JD, Wong J, Alders R. Effective animal health programming requires consideration of and communication with those at the human-animal interface. Perspect Agric Vet Sci Nutr Nat Resour. 2016;11:1–7. 10.1079/PAVSNNR201611030

[39] Moore TC, Fong J, Rosa Hernández AM, Pogreba-Brown K. CAFOs, novel influenza, and the need for One Health approaches. One Health. 2021;13:100246. 10.1016/j.onehlt.2021.100246 33997233 PMC8091921

[40] Wong JT, Bagnol B, Grieve H, da Costa Jong JB, Li M, Alders RG. Factors influencing animal-source food consumption in Timor-Leste. Food Secur. 2018;10:741–62. 10.1007/s12571-018-0804-5

[41] Food and Agriculture Organization of the United Nations. Hunger and food insecurity [cited 2023 Jan 28]. https://www.fao.org/hunger/en

[42] World Health Organization. Quadripartite memorandum of understanding (MoU) signed for a new era of One Health collaboration. 2022 Apr 29 [cited 2023 Jan 28]. https://www.who.int/news/item/29-04-2022-quadripartite-memorandum-of-understanding-(mou)-signed-for-a-new-era-of-one-health-collaboration

[43] Food and Agriculture Organization of the United Nations, United Nations Environment Programme, World Health Organization, World Organisation for Animal Health. One Health joint plan of action (2022–2026): towards a more comprehensive One Health, approach to global health threats at the human-animal-environment interface. Rome: Food and Agriculture Organization of the United Nations, United Nations Environment Programme, World Health Organization, World Organisation for Animal Health; 2022.

[44] Rulli MC, D’Odorico P, Galli N, Hayman DTS. Land-use change and the livestock revolution increase the risk of zoonotic coronavirus transmission from rhinolophid bats. Nature Food. 2021;2:409–16. 10.1038/s43016-021-00285-x 37118224

[45] Alders RG, Bagnol B, Young MP. Technically sound and sustainable Newcastle disease control in village chickens: lessons learnt over fifteen years. Worlds Poult Sci J. 2010;66:433–40. 10.1017/S0043933910000516

[46] Ayala AJ, Yabsley MJ, Hernandez SM. A review of pathogen transmission at the backyard chicken–wild bird interface. Front Vet Sci. 2020;7:539925. 10.3389/fvets.2020.539925 33195512 PMC7541960

[47] Miller PJ, Torchetti MK. Newcastle disease virus detection and differentiation from avian influenza. In: Spackman E, editor. Animal influenza virus. New York: Springer; 2014. p. 235–9. 10.1007/978-1-4939-0758-8_19 24899433

[48] Smith D, Cooper T, Pereira A, Jong JBDC. Counting the cost: The potential impact of African Swine Fever on smallholders in Timor-Leste. One Health. 2019;8:100109. 10.1016/j.onehlt.2019.100109 31687470 PMC6819879

[49] World Health Organization. Reducing public health risks associated with the sale of live wild animals of mammalian species in traditional food markets [cited 2023 Jan 28]. https://www.who.int/publications/i/item/WHO-2019-nCoV-Food-safety-traditional-markets-2021.1

[50] Koh LP, Li Y, Lee JSH. The value of China’s ban on wildlife trade and consumption. Nat Sustain. 2021;4:2–4. 10.1038/s41893-020-00677-0

[51] McCall R. Eating bats and pangolins banned in Gabon as a result of coronavirus pandemic. Newsweek. 2020 Apr 6 [cited 2023 Jan 28]. https://www.newsweek.com/eating-bats-pangolins-gabon-coronavirus-pandemic-1496329

[52] Xiao L, Lu Z, Li X, Zhao X, Li BV. Why do we need a wildlife consumption ban in China? Curr Biol. 2021;31:R168–72. 10.1016/j.cub.2020.12.036 33621498 PMC8860476

[53] Xiao X, Newman C, Buesching CD, Macdonald DW, Zhou Z-M. Animal sales from Wuhan wet markets immediately prior to the COVID-19 pandemic. Sci Rep. 2021;11:11898. 10.1038/s41598-021-91470-2 34099828 PMC8184983

[54] Berthe FCJ, Bouley T, Karesh WB, Le Gall FG, Machalaba CC, Plante CA, et al. One Health: operational framework for strengthening human, animal, and environmental public health systems at their interface. Washington: World Bank; 2018.

[55] Watsa M; Wildlife Disease Surveillance Focus Group. Rigorous wildlife disease surveillance. Science. 2020;369:145–7. 10.1126/science.abc0017 32646989

[56] Machalaba CC, Salerno RH, Barton Behravesh C, Benigno S, Berthe FCJ, Chungong S, et al. Institutionalizing One Health: from assessment to action. Health Secur. 2018;16(S1):S37–43. 10.1089/hs.2018.0064 30480500

[57] Kuisma E, Olson SH, Cameron KN, Reed PE, Karesh WB, Ondzie AI, et al. Long-term wildlife mortality surveillance in northern Congo: a model for the detection of Ebola virus disease epizootics. Philos Trans R Soc Lond B Biol Sci. 2019;374:20180339. 10.1098/rstb.2018.0339 31401969 PMC6711308

[58] Eby P, Peel AJ, Hoegh A, Madden W, Giles JR, Hudson PJ, et al. Pathogen spillover driven by rapid changes in bat ecology. Nature. 2023;613:340–4. 10.1038/s41586-022-05506-2 36384167 PMC9768785

[59] Epstein JH, Anthony SJ. Viral discovery as a tool for pandemic preparedness. Rev Sci Tech. 2017;36:499–512. 10.20506/rst.36.2.2669 30152468

[60] Br A, Dovih P, Ramakrishnan U, Liang E, Mendenhall I, Hong DLW, et al. Evidence of filovirus and henipavirus in bats and bat harvesters, India. Int J Infect Dis. 2019;79:60. 10.1016/j.ijid.2018.11.156

[61] Wong EB, Olivier S, Gunda R, Koole O, Surujdeen A, Gareta D, et al.; Vukuzazi Team. Convergence of infectious and non-communicable disease epidemics in rural South Africa: a cross-sectional, population-based multimorbidity study. Lancet Glob Health. 2021;9:e967–76. 10.1016/S2214-109X(21)00176-5 34143995 PMC8220132

[62] Maze MJ, Bassat Q, Feasey NA, Mandomando I, Musicha P, Crump JA. The epidemiology of febrile illness in sub-Saharan Africa: implications for diagnosis and management. Clin Microbiol Infect. 2018;24:808–14. 10.1016/j.cmi.2018.02.011 29454844 PMC6057815

[63] Shear MD, Goodnough A, Kaplan S, Fink S, Thomas K, Weiland N. The lost month: how a failure to test blinded the U.S. to Covid-19. The New York Times. 2020 Mar 28 [cited 2023 Jan 28]. https://www.nytimes.com/2020/03/28/us/testing-coronavirus-pandemic.html

[64] Lewis TUS. Monkeypox response has been woefully inadequate, experts say. Scientific America. 2022 Jul 14 [cited 2023 Jan 28]. https://www.scientificamerican.com/article/monkeypox-testing-and-vaccination-in-u-s-have-been-vastly-inadequate-experts-say1

[65] Sánchez CA, Li H, Phelps KL, Zambrana-Torrelio C, Wang L-F, Zhou P, et al. A strategy to assess spillover risk of bat SARS-related coronaviruses in Southeast Asia. Nat Commun. 2022;13:4380. 10.1038/s41467-022-31860-w 35945197 PMC9363439

[66] Borrega R, Nelson DKS, Koval AP, Bond NG, Heinrich ML, Rowland MM, et al. Cross-reactive antibodies to SARS-CoV-2 and MERS-CoV in pre-COVID-19 blood samples from Sierra Leoneans. Viruses. 2021;13:2325. 10.3390/v13112325 34835131 PMC8625389

[67] PREDICT Consortium. Advancing global health security at the frontiers of disease emergence. Davis (CA): One Health Institute, University of California, Davis; 2020 [cited 2022 Nov 30]. https://ohi.vetmed.ucdavis.edu/sites/g/files/dgvnsk5251/files/inline-files/PREDICT%20LEGACY%20-%20FINAL%20FOR%20WEB%20-compressed_0.pdf

[68] McKinsey & Company. Not the last pandemic: Investing now to reimagine public-health systems. 2021 [cited 2021 Nov 1]. https://www.mckinsey.com/industries/public-and-social-sector/our-insights/not-the-last-pandemic-investing-now-to-reimagine-public-health-systems

[69] Gruetzmacher K, Karesh WB, Amuasi JH, Arshad A, Farlow A, Gabrysch S, et al. The Berlin principles on one health - Bridging global health and conservation. Sci Total Environ. 2021;764:142919. 10.1016/j.scitotenv.2020.142919 33097250 PMC7550087

[70] Alders RG, Chadag MV, Debnath NC, Howden M, Meza F, Schipp MA, et al. Planetary boundaries and veterinary services. Rev Sci Tech. 2021;40:439–53. 10.20506/rst.40.2.3236 34542103

